# Ten Good Reasons to Practice Neuroultrasound in Critical Care Setting

**DOI:** 10.3389/fneur.2021.799421

**Published:** 2022-01-13

**Authors:** Carla Bittencourt Rynkowski, Juliana Caldas

**Affiliations:** ^1^Intensive Care Unit of Cristo Redentor Hospital, Porto Alegre, Brazil; ^2^Intensive Care Unit, Hospital Ernesto Dornelles, Porto Alegre, Brazil; ^3^Escola Bahiana de Medicina e Saúde Pública, Salvador, Brazil; ^4^Instituto D'Or de Pesquisa e Ensino (IDOR), Salvador, Brazil

**Keywords:** transcranial Doppler, ultrasonography, intensive care unit, optic nerve sheath, transcranial color-coded duplex, neurocritical care

## Abstract

In the beginning, cerebral ultrasound (US) was not considered feasible because the intact skull was a seemingly impenetrable obstacle. For this reason, obtaining a clear image resolution had been a challenge since the first use of neuroultrasound (NUS) for the assessment of small deep brain structures. However, the improvements in transducer technologies and advances in signal processing have refined the image resolution, and the role of NUS has evolved as an imaging modality for the brain parenchyma within multiple pathologies. This article summarizes ten crucial applications of cerebral ultrasonography for the evaluation and management of neurocritical patients, whose transfer from and to intensive care units poses a real problem to medical care staff. This also encompasses ease of use, low cost, wide acceptance by patients, no radiation risk, and relative independence from movement artifacts. Bedsides, availability and reliability raised the interest of critical care intensivists in using it with increasing frequency. In this mini-review, the usefulness and the advantages of US in the neurocritical care setting are discussed regarding ten aspects to encourage the intensivist physician to practice this important tool.

## Introduction

Ultrasound (US) has gained a prominent role in critical care clinical practice. Cerebral US does not replace advanced and precise static imaging exams, it adds dynamic cerebrovascular information of a non-invasive test at the bedside, that does not displace the patient from the intensive care unit (ICU), does not expose them to radiation, and allows for prompt re-evaluation after some intervention or clinical change (1, 2). Recent consensus has been published, such as neuroultrasound (NUS) in basic US skills that intensivists should possess/acquire for the evaluation and management of critically ill patients (3).

We propose the performance of NUS that comprises US measurement of the optic nerve sheath diameter (ONSD), transcranial Doppler (TCD) evaluation by the “conventional” or “blind” method, and transcranial color-coded duplex (TCCD). This study presents 10 reasons why NUS should be part of the current clinical practice dealing with ICU patients, considering different clinical contexts of patients admitted to a mixed general-neuro ICU.

### Non-invasive Intracranial Pressure: The First and Legitimate One

Intracranial hypertension (IH) is one of the most hazardous situations in neurocritical care, an emergency that can occur in severe traumatic brain injury (TBI), extensive ischemia of stroke or subarachnoid hemorrhage (SAH), the mass effect of intracranial hemorrhage or another intracranial lesion, such as tumor, abscess, and even advanced cerebral edema. It needs prompt recognition and management (3). Not all patients will have access to invasive intracranial pressure measurement, the gold-standard method to verify IH (4).

**Figure 1:**
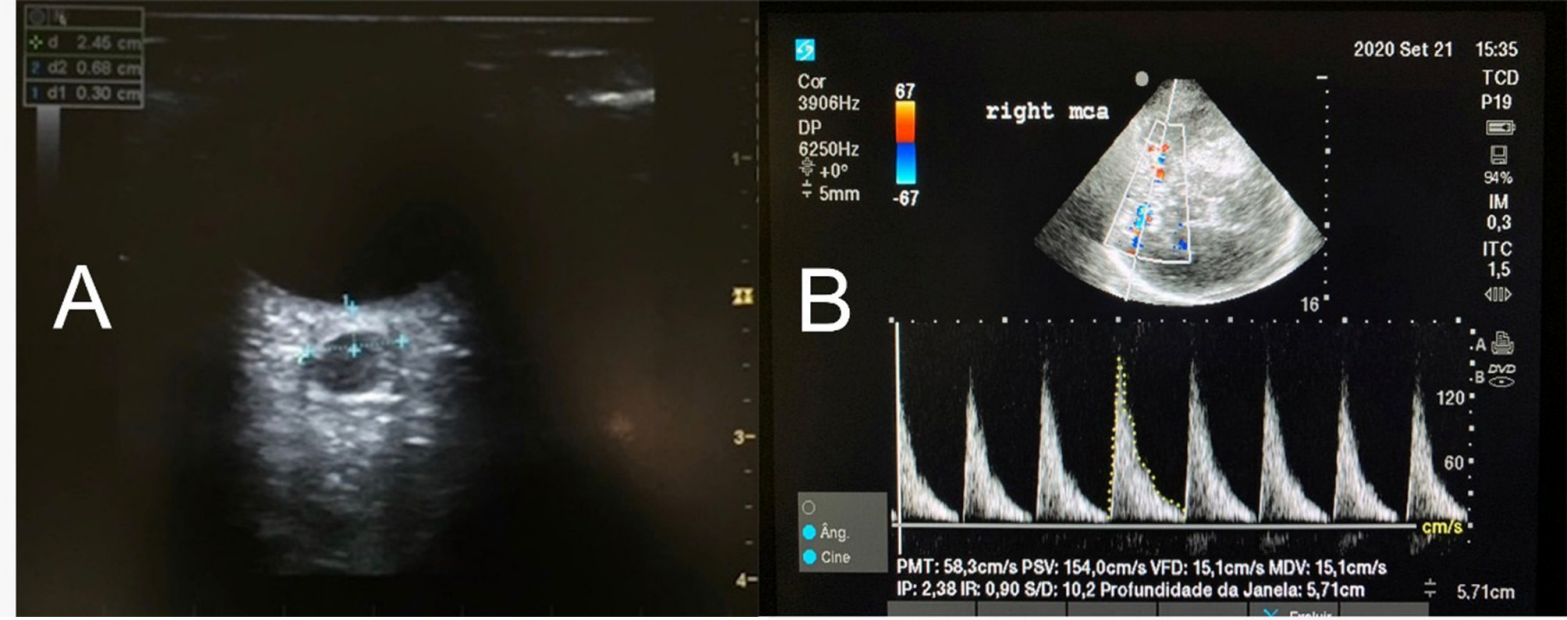
**(A)** The axial image of the optic nerve sheath diameter (ONSD) in a patient with increased ICP. The ONSD is measured with a linear transducer placed over the closed eye and the measurement of the ONSD is performed perpendicularly to an electronic caliper positioned 3 mm behind the retina. The same measurement is performed at least 3 times (or more, if the variation in between is beyond 10%). Each eye will have a mean value. **(B)** MCA waveform with color Doppler. PSV, Peak systolic velocity, in cm/s. VDV, End diastolic velocity, in cm/s. Note the Pulsatility Index (IP) suggesting high ICP.

Considering the non-invasive assessment of IH in the US modality, the simplest one is the measurement of ONSD (4). An enlarged ONSD (Figure 1A) may correspond to HI. Both sides should be measured rather in a sequential comparison. ONSD can enlarge a few minutes after IH begins (2, 5, 6) and also decrease minutes after IH control. Studies are not uniform about a precise cut-off to be considered enlarged ONSD. A meta-analysis has demonstrated a wide range of diagnostic odds ratio [67.5 (95% CI 29–135)] of sonographic ONSD measurement to detect IH (7). Even though, diameters larger than 5–6 mm in adults are currently considered enlarged. In current clinical practice, measurement of ONSD is a tool to alert about IH suspicion or discard it (2).

Transcranial color-coded duplex and TCD are methods based on cerebral blood flow (CBF) velocity that are related to cerebral perfusion pressure (CPP). In IH and compromised CPP, there is a decrease in diastolic flow velocity (Figure 1B) and consequently an increase in the pulsatility index (PI) (systolic flow velocity-diastolic flow velocity/mean flow velocity [MFV]). Diastolic flow velocity near zero and PI values higher than 2 are strongly suggestive of IH and low CPP (Figure 1B) (8). Studies have described a good correlation between invasive CPP and non-invasive CPP (estimated by the formula: nCPP = mean arterial pressure × diastolic flow velocity/MFV + 14 mmHg) (*R* = 0.61; *p* = 0.003) (2, 9). A recent study has demonstrated that TCD values combined with ONSD measurement increased the accuracy [0.91 (0.84–0.97)] of non-invasive methods to detect IH (9).

### Second and Paramount: Diagnosing Cerebral Circulatory Arrest (CCA)

In many countries, it is necessary to use an image complementary diagnostic tool besides a clinical exam for the legal diagnosis of brain death (BD). TCD is a common method recognized by this application. Brunser et al. (10) demonstrated a sensitivity and specificity for the diagnosis of CCA of 100 and 98%, respectively, and the positive and negative likelihood ratios for BD were 45 and 0, respectively. Besides, the TCD method has the advantages of not moving the patient from ICU and not using contrast.

Before performing the TCD exam for CCA diagnosis, the systolic arterial pressure must be at least 70 mmHg to guarantee that this cerebral circulatory failure is not attributed to hemodynamics conditions (10). The absence of CBF is then confirmed by the presence of any of the 4 patterns in both MCA and vertebral arteries: diastolic blunting (diastolic flow velocity becomes zero), diastolic flow reversal or biphasic flow (diastolic flow, which was previously zero, appears at the opposite direction, indicating the retrograde flow of the diastolic phase in the cardiac cycle), systolic flow spikes (minimal spikes that correspond to cardiac systole), and no TCD signal. If there is no TCD signal, it should have a previous detection of TCD signal in the same acoustic window, performing TCD at least 2 times within 30-min apart (2, 10).

### Monitoring Cerebral Hemodynamic

Transcranial Doppler-based methods allow the detection of cerebral circulation status through the analysis of CBF velocities intermittently or continuously. Besides recognizing critical patrons of CBF, compromised CPP, and IH, it can also be found oligemia, hyperemia, and vasospasm (2). Those transcranial findings can be common to different pathologies.

In acute stroke, TCD or TCCD can detect stenosis, large vessels occlusion, hemorrhagic complication, recanalization, and patrons of hypoperfusion and hyperperfusion that deserves increased attention if CA is impaired. NUS can also detect malignant infarction and early cerebral perfusion compromise to be submitted to an early decompressive craniectomy (DC) (2).

In patients with TBI, besides IH, there are some characteristic sonographic findings associated with unfavorable outcomes. According to Martin et al., there are 3 sonographic phases that can appear during the first days after TBI (11). On the first day, it is expected oligemia with low CBF velocities. In the next 3 days, hyperemia, with high velocity but not as high as vasospasm. Moreover, in the next 10 d, vasospasm. All these patrons can appear in a different order during the days after TBI. The presence of any of these 3 patrons is related to the development of unfavorable outcomes. Also, the presence of low diastolic flow velocity and high pulsatility index is related to unfavorable outcomes (12).

After DC, when the initial insult is resolved, an oligemic patron is observed ipsilateral the skull defect while normal velocities are observed on the opposite side. Besides, right after cranioplasty, there is a normalization of those previous low CBF velocities (13–16). The physical phenomenon that justifies these cerebral hemodynamic differences is not completely understood, but we must bear it in mind while proceeding with this evaluation.

### Assessing Vasospasm, the Forth, and Nice One

In 2012, the American Heart Association guidelines for the management of SAH recommended the use of TCD for monitoring the development of arterial vasospasm with class IIa/level B evidence, and this setting is one of the most common and current applications of TCD use. Vasospasm corresponds to a vessel narrowing and should not be used as a synonym of clinical neurological deterioration in DCI (17), nevertheless, TCD-detected vasospasm is significantly predictive of Delayed cerebral ischemia in current studies.

For the middle cerebral artery (MCA), MFVs of <120 cm/s or >200 cm/s, a rapid rise in flow velocities or a higher Lindegaard Index (LI = MFV of MCA/MFV of ipsilateral internal carotid) may predict the absence or presence of clinically significant angiographic MCA vasospasm (12, 17). An MFV <120 cm/s has a 94% negative predictive value, while an MFV > 200 cm/s has an 87% positive predictive value. MCA vasospasm is graded as: mild, MFV > 120–150 cm/s mild, MFV > 120–150 cm/s or LR 3.0–4.5; moderate, MFV > 150–200 cm/s or LR 4.5–6.0; severe, MFV > 200 cm/s or LR > 6.0, or LR 3.0–4.5; moderate, MFV > 150–200 cm/s or LR 4.5–6.0; severe, MFV > 200 cm/s or LR >6.0. For the basilar artery (BA), BA/extracranial vertebral artery ratio >2 (Soustiel index) was associated with 73% sensitivity and 80% specificity for BA vasospasm (8).

In hyperemia, the MFVs of both intracerebral and extracerebral vessels are elevated (LI <3). After the rapid increase of MFV at the onset of vasospasm, there is a slow daily MFV reduction, around 6 cm per second per day (18). TCD detects vasospasm when artery narrowing is over 25%, being arteriography more sensitive than TCD to detect vasospasm (19).

The sonographic vasospasm diagnosis has special importance when the neurological exam is compromised in poor-grade patients or sedated patients. Guidelines in SAH recommend TCD to be performed around every 48 h at least during the first 14 d, the risky period to develop vasospasm (19).

In SAH, besides vasospasm, NUS findings can suggest IH, hydrocephalus, rebleeding, and cerebral infarction. NUS investigation can help to set the best moment to insert a ventricular shunt and proceed with a DC (2).

### Investigating Shunt

In patients with ischemic stroke or transient ischemic attack, an early diagnostic evaluation is recommended for gaining insights into the etiology of stroke and the planning of optimal strategies to prevent recurrent stroke (20). TCD tool allows early evaluation of the presence of patent foramen ovale (PFO) and/or intrapulmonary arteriovenous shunt, still within ICU stay (21).

To detect microbubbles by TCD, a gaseous contrast agent is injected into the peripheral vein or agitated saline bubble (10-ml syringe filled with 9 ml of saline solution and 1 ml of air, and microbubbles are produced by shaking the syringe). The microbubbles pass from the right to the left circulation during the cardiac cycle, enter the systemic circulation, and TCD picks them up (9). TCD should be performed during normal breathing and repeated in association with provocative maneuvers, such as the Valsalva maneuver, and successive coughs to increase the accuracy of the procedure (22) (Supplementary Material).

However, transesophageal echocardiography is considered the gold standard for PFO diagnosis. It is a semi-invasive technique that relies on the compliance of the patient to a great extent and could not always detect latent shunts either. On the other hand, a pooled analysis of a systematic literature review found that TCD had 96.1% sensitivity (95% CI, 93.0–97.8) and 92.4% specificity (95% CI, 85.5–96.1) compared to transesophageal echocardiography for detection of right-to-left shunting (22). Besides this, it is a more sensitive tool for detecting a shunt at the non-cardiac level (i.e., patent ductus arteriosus) (23).

### Sequential Cerebral US—A Helpful Tool in Evaluating Hydrocephalus

Hydrocephalus is a devastating complication, and it can occur in up to 30% of patients with SAH, but also in intracranial hemorrhagic and/or mass lesions of the posterior fossa and acute meningeal diseases (1, 2). Sequential non-invasive bedside monitoring of the ventricular system is crucial in disturbances of cerebrospinal fluid circulation, whereas part of them is comatose or sedated, and clinical examination is not the easiest method to monitor for hydrocephalus.

**Figure 2:**
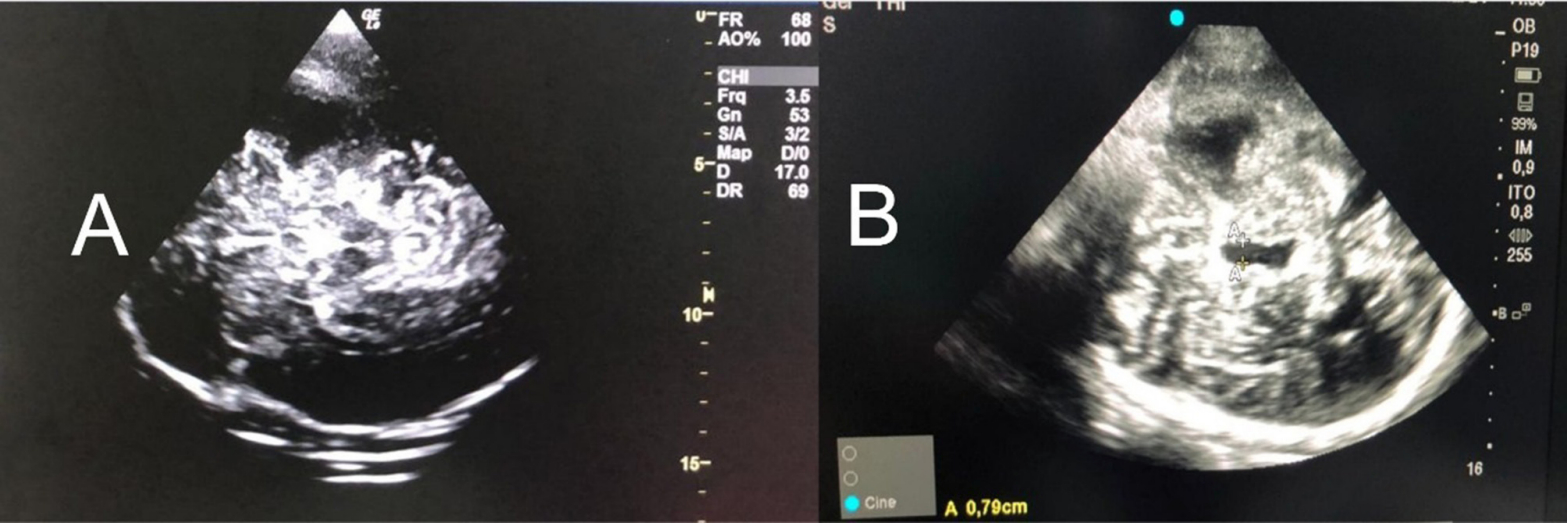
**(A)** Mesencephalic plane and the imaging of the butterfly-shaped brainstem is a prerequisite to obtain a landmark for orientation [observed in 90–95% of the patients (3–5)]. **(B)** Tilting the probe 10° cranially, in a diencephalic plane and the third ventricle is obtained (A-A). It should be measured the largest transverse diameter of the third ventricle with its hyperechogenic margins. In addition, the frontal horn of the contralateral lateral ventricle can be measured (5). To ensure the accurate and reproducible measurement of the ventricles' widths, measurements with the US should be performed from the ipsilateral to the contralateral inner layer of the hyperechogenic ependyma. US, ultrasound.

Transcranial color-coded duplex section should start from the mesencephalic plane, and the imaging of the butterfly-shaped brainstem is a prerequisite to obtain a landmark for orientation, which can be observed in 90–95% of the patients (7, 23, 24). The largest transverse diameter of the third ventricle with its hyperechogenic margins is imaged by tilting the duplex beam approximately 10 degrees upward (Figure 2A). At this plane, the transverse diameter of the third ventricle and of the frontal horn of the contralateral lateral ventricle can be measured (25). To ensure the accurate and reproducible measurement of the ventricles' widths, measurements with the US should be performed from the ipsilateral to the contralateral inner layer of the hyperechogenic ependyma (Figure 2B). Because the diameter of the ventricles, particularly the lateral ones, depends on the angle of the probe, the correlation between NUS and CT measurements is higher for the widths of the third ventricles than for the lateral ventricles (25).

Transcranial color-coded duplex can be used to follow hydrocephalus (3, 6) and is considered a reliable technique to predict the need for cerebrospinal fluid drainage in patients with external ventricular drainage (EVD). It estimated that a cut-off value of an increase of 5.5 mm in ventricle width after clamping had high sensitivity (100%) and negative predictive value (100%) (3).

### Detecting and Monitoring Intracranial Hematomas

Neuroultrasound in intracranial hemorrhage can contribute with information about the hematoma *per se*, but also about the cerebral hemodynamics repercussions of its presence. TCCD can detect the hyperechogenic aspect of an intracranial hematoma, specially up to 3 d after hemorrhage but rarely after 14 days (10). It also estimates hematoma volume and monitors its expansion and midline shift, identifying hydrocephalus and hemorrhagic hydrocephalus, besides detecting a possible CPP compromise. ONSD measurement can identify those patients whose hematoma is causing IH, generally expected for hematomas larger than 25 ml (2). An initial NUS evaluation and daily hematoma monitoring measurement are recommended to early detect possible hematoma expansion and associated complications (2). Those patients who expand intracerebral hematoma or rebleed present an increased risk of developing an unfavorable outcome (10).

### Evaluation the Cerebral Autoregulation (CA) in Critical Care Patients: Is There a Place?

Cerebral autoregulation defines the brain's capacity to provide stable CBF despite fluctuations in mean arterial blood pressure (BP) (26). CA assessments are generally classified as “static” or “dynamic” and can be performed by measuring the CBF velocity of the MCA with TCD, simultaneously with BP (27). Static CA represents the CBF dependence on BP under steady-state conditions (27), and dynamic CA reflects the transient response of CBF to sudden fluctuations in BP.

The relevance of CA assessment is increasingly recognized in critical care scenarios. CA impairment can predict unfavorable outcomes in TBI and SAH (28, 29). In non-neurological patients, such as those who underwent cardiac surgery, the assessment of CA before and after surgery has the potential for early identification of patients at risk of delirium (29). In sepse, patients can present impaired CA. Circulatory shock patients were often associated with impairment of CA and the severity of CA alterations correlated with the degree of multiple organ failures (30).

There are several indexes and methods in the literature and no specific index is currently considered to be the “gold standard”. The autoregulation index (ARI) and the mean flow index (Mxa) (31) have been the ones most used once they do not require invasive cerebral monitoring (32). Both indexes are based on spontaneous fluctuation in BP and their correlation with changes in CBF velocity, measured by TCD. The ARI is derived from spontaneous fluctuations in BP and CBF velocity, and it reflects both the temporal and amplitude relations between CPP and flows, which characterizes the dynamic CA approach (33). ARI = 0 indicates absence of CA, while ARI = 9 corresponds to the most efficient CA. Mxa, assessed within the framework of linear regression analysis, also using spontaneous fluctuations of BP, is a “quasi-static” approach since in most cases no information can be obtained about the speed of the response (32). Impaired CA was defined as Mxa > 0.3.

An elegant technique to assess static CA at the bedside that does not require additional software is the transient hyperemic response test (THRT) with TCD. It investigates changes in peak flow velocity (PFV) in ACM after a brief compression (reduction ≥30% of the baseline PFV) of the ipsilateral common carotid artery (3–5 s) (34). Each side should be tested separately. CA is preserved when a hyperemic response occurs (an increase >9%: THRT ratio ≥ 1.09) on both sides (35). In SAH, different CA can predict functional outcome (36).

The study of CA in TBI has advanced. It is possible to estimate the ideal arterial pressure range at which patients should be managed in the acute phase (37). Studies have demonstrated that those patients who kept a range of individualized CPP goals developed a better outcome than those managed according to a general value of current guidelines (37). The calculation of the optimal CPP is based on the continuous monitoring of PRx (CA index of the correlation between arterial BP and intracranial pressure).

Moreover, in patients with ICH who need strict arterial pressure control to avoid hypertension, it is very important to know about the CA status. Patients that present failure of CA are at risk of presenting cerebral hyper or hypoflow according to arterial pressure oscillations (38).

### Appraising Cerebral the Hemodynamic After Reperfusion Treatments

Patients who underwent reperfusion treatments, such as thrombectomy after ischemic stroke and carotid endarterectomy, have a risk of bleeding and cerebral hyperperfusion syndrome. BP control plays an important role in the management of these patients in the ICU setting.

In these patients, TCD can demonstrate cerebral hemodynamic status after these reperfusion procedures and avoid hyperemia or oligemia, which are linked to brain edema and IH. We still cannot set an individualized BP goal for those patients submitted to thrombolytic, thrombectomy, or endarterectomy, considering the risk of hemorrhagic transformation (39). The current guidelines suggest the BP to be <185 mmHg systolic and <110 mmHg diastolic before treatment with thrombolytic and <180/105 mmHg for the first 24 h after treatment (39). Other studies recommend lower systolic BP to be <140 mmHg after optimal recanalization is achieved. Nonetheless, it is unknown the exact BP at a lower risk of hemorrhage and edema after these therapies.

The analysis of MCA can avoid hyperemia state (risk of hemorrhagic transformation), studies have been shown that a 1.5-fold postoperative increase of MCA MFV compared with preoperative levels may predict the occurrence of cerebral edema (39). TCD can individualize early management in the ICU point-of-care.

### Last One, but Extremely Useful: Detecting Brain Midline Shift

Cerebral midline shift (MLS) is a life-threatening condition that requires urgent diagnosis and treatment. In cases of a cerebral, CT-scan is not immediately available and ICP could potentially raise during transport of the patient, NUS has been proposed as a useful tool to estimate MLS at the bedside (2, 25, 40).

Recent systematic review and meta-analysis were performed to assess the reliability of NUS to measure MLS when compared with cerebral CT-scan, 10 studies, such as 416 patients and 492 examinations, were analyzed and the authors concluded that NUS may be a reliable alternative to brain imaging for the rapid evaluation of cerebral MLS in brain-injured patients (7).

## Conclusion

Although the main application of point-of-care US involves primarily the investigation of the chest, abdomen, vessels, cerebral US presents a huge contribution to neurocritical patient evaluation and could also be integrated into critical care scenarios. More than a single and isolated exam, these methods should be added to the routine of clinical practice with neurocritical patients.

## Author Contributions

CBR and JC contributed to concept and design of the topic. CBR wrote sessions Non-Invasive Intracranial Pressure: the First and Legitimate One, Second and Paramount: Diagnosing Cerebral Circulatory Arrest (CCA), Monitoring Cerebral Hemodynamic, and Assessing Vasospasm, the Forth, and Nice One. JC wrote sessions Investigating Shunt, Sequential Cerebral US–a Helpful Tool in Evaluating Hydrocephalus, Detecting and Monitoring Intracranial Hematomas, Evaluation the Cerebral Autoregulation (CA) in Critical Care Patients: Is There a Place?, Appraising Cerebral the Hemodynamic After Reperfusion Treatments, and Last One, but Extremely Useful: Detecting Brain Midline Shift. All authors contributed to the article and approved the submitted version.

## Conflict of Interest

The authors declare that the research was conducted in the absence of any commercial or financial relationships that could be construed as a potential conflict of interest.

## Publisher's Note

All claims expressed in this article are solely those of the authors and do not necessarily represent those of their affiliated organizations, or those of the publisher, the editors and the reviewers. Any product that may be evaluated in this article, or claim that may be made by its manufacturer, is not guaranteed or endorsed by the publisher.

## Supplementary Material

The Supplementary Material about microbubbles test by TCD for this article can be found online at: https://www.frontiersin.org/articles/10.3389/fneur.2021.799421/full#supplementary-material
